# Evaluating Outcomes in Patients with Metabolic Dysfunction-Associated Steatotic Liver Disease and Vitamin D Deficiency

**DOI:** 10.3390/diseases14070243

**Published:** 2026-07-04

**Authors:** Tiana Dodd, Arpit Sharma, Nisar Amin, Veysel Tahan, Ebubekir Daglilar, Nikki Duong

**Affiliations:** 1 Charleston Area Medical Center, Charleston, WV 25304, USA; nisar.amin@vandaliahealth.org (N.A.); veysel.tahan@vandaliahealth.org (V.T.); ebubekir.daglilar@vandaliahealth.org (E.D.); 2 Monash Health, Melbourne, VIC 3168, Australia; cobater.30.2002@gmail.com; 3 Internal Medicine-Gastroenterology, West Virginia University, Morgantown, WV 25304, USA; 4 Division of Gastroenterology and Hepatology, Stanford University, Standford, Palo Alto, CA 94304, USA; nduong91@stanford.edu

**Keywords:** MASLD, chronic liver disease, vitamin D deficiency

## Abstract

**Background:** Metabolic dysfunction-associated steatotic liver disease (MASLD) is the leading cause of chronic liver disease (CLD) globally and is closely linked to metabolic risk factors and systemic inflammation. Emerging evidence suggests that vitamin D deficiency may influence MASLD severity and outcomes, though limited real-world data often assess long-term clinical outcomes in MASLD patients stratified by vitamin D status. **Methods:** We conducted a retrospective cohort study using the TriNetX US Collaborative Network (2006–2025). Adult patients with MASLD were stratified into two cohorts based on serum 25-hydroxyvitamin D levels: normal (≥30 ng/mL) and deficient (<20 ng/mL). Patients with other CLD, malignancy, decompensated cirrhosis, and relevant confounding conditions were excluded. Primary outcomes included all-cause mortality, hospital readmissions, and ICU admissions at 1-year and 5-year follow-up. **Results:** After propensity score matching, 6959 patients were included in each cohort. Compared with patients with normal vitamin D levels, those with vitamin D deficiency had significantly higher rates of hospital readmissions, ICU admissions, and all-cause mortality at both 1-year and 5-year follow-up. A 1 year, readmissions occurred in 10% vs. 6%, ICU admissions 2.6% vs. 1.2%, and mortality 1.5% vs. 0.5% of patients (*p* = 0.01). Similar findings were observed at 5 years, with higher rates of readmissions 15% vs. 10%, ICU admissions 4.4% vs. 2.4% and mortality 3.2% vs. 1.3% in the vitamin D-deficient cohort (*p* = 0.01). **Conclusions:** Vitamin D deficiency was associated with significantly increased mortality, hospital readmissions, and ICU admissions among patients with MASLD. Our findings suggest that vitamin D status may represent a valuable prognostic indicator in this population. Although the observational nature of this study precluded establishing causality, our results support the consideration of routine assessment of vitamin D levels in patients with MASLD. Further prospective and mechanistic studies are needed to determine whether vitamin D supplementation can improve outcomes in this population.

## 1. Introduction

Metabolic dysfunction-associated steatotic liver disease (MASLD) is the most prevalent cause of chronic liver disease (CLD) worldwide, affecting more than 30% of the global population and representing a growing public health burden [1]. MASLD is characterized by the presence of hepatic steatosis on imaging in conjunction with at least one cardiometabolic risk factor, including BMI ≥ 25 kg/m^2^, type 2 diabetes mellitus, hypertension, hypertriglyceridemia, or low high-density lipoprotein (HDL) cholesterol levels < 40 or treatment with a lipid-lowering agent [2]. The pathogenesis of MASLD is closely linked to metabolic dysfunction, insulin resistance, and chronic systemic inflammation, which contribute to disease progression and the development of hepatic fibrosis.

Accumulating evidence suggests that the burden of metabolic risk factors plays a critical role in determining disease severity and clinical outcomes. In a large cohort study, the risk of advanced fibrosis and MALSD-related mortality increased progressively with the number of cardiometabolic risk factors present, highlighting the impact of metabolic dysfunction on liver disease progression [3]. The relationship between metabolic risk factors and MASLD progression is multifactorial and driven by a complex interplay between metabolic and inflammatory pathways. Proposed mechanisms include insulin resistance, dyslipidemia, oxidative stress, and chronic low-grade systemic inflammation [4]. These pathophysiologic processes not only promote liver disease progression but also increase the risk of extrahepatic complications.

The global prevalence of MASLD among adults is around 30%, with a steady rise of nearly 1% per year over the last three decades [1]. This trend parallels the global epidemic of obesity and type 2 diabetes, which are key drivers of disease development. The prevalence of MASLD varies considerably across geographic regions, with the highest rates observed in Latin America and the Middle East, and lower prevalence reported in Western Europe [5,6]. These regional differences likely represent heterogeneity in genetic predisposition, dietary composition, healthcare access, physical activity patterns, and background prevalence of metabolic syndrome. Current projections estimate that MASLD may affect nearly one-half of the adult population by 2050, underscoring its growing impact on healthcare systems and its emergence as a major global public health challenge [7].

MASLD is increasingly recognized as a multisystem disorder characterized by complex metabolic, inflammatory, and endocrine dysregulation. The disease exists along a spectrum ranging from simple hepatic steatosis to more advanced stages of liver injury. When hepatic steatosis is accompanied by hepatocellular ballooning and lobular inflammation, the condition is classified as metabolic dysfunction-associated steatohepatitis (MASH), the progressive form of MASLD associated with an increased risk of fibrosis and adverse clinical outcomes [8]. Although many patients remain stable with isolated steatosis, approximately 10–30% will progress to MASH over time [8,9]. Disease progression is driven by a complex interplay of insulin resistance, oxidative stress, lipotoxicity, and chronic systemic inflammation, which promote hepatocellular injury and fibrogenesis.

The development of hepatic fibrosis is the strongest predictor of liver-related morbidity and mortality in MASLD. Longitudinal studies have demonstrated that approximately 15% of patients with MASH and early-stage fibrosis progress to cirrhosis and/or hepatic decompensation within 8–13 years [10,11,12]. As fibrosis advances, patients face an increased risk of cirrhosis, portal hypertension, hepatic decompensation, liver failure, and hepatocellular carcinoma, resulting in substantial healthcare utilization and reduced survival.

Beyond its hepatic manifestations, MASLD is closely linked to a broad range of extrahepatic complications, including cardiovascular disease, type 2 diabetes mellitus, chronic kidney disease, and metabolic syndrome, all of which contribute significantly to overall morbidity and mortality [13]. Cardiovascular disease remains one of the leading causes of death among patients with MASLD, emphasizing the systemic nature of the disease and the importance of comprehensive management strategies that address both hepatic and extrahepatic risk factors [14,15].

The progression of MASLD is currently understood through the “multiple-hit” hypothesis, wherein the hepatic steatosis driven by insulin resistance mentioned above acts as the primary “hit.” This initial metabolic stress sensitizes the liver to a cascade of secondary insults, including oxidative stress, metabolic factors and gut-derived endotoxemia [16]. Vitamin D deficiency potentially acts as a crucial “hit” in this sequence, as the loss of its protective signaling may accelerate the transition from simple steatosis to MASH and irreversible fibrosis. As such, more attention has been directed to identifying additional metabolic factors that may modify disease severity or progression. Emerging evidence further suggests a potential role of vitamin D deficiency in the pathogenesis and clinical course of MASLD.

Several studies have demonstrated an inverse relationship between serum vitamin D levels and BMI, with lower vitamin D concentrations being associated with an increased prevalence and severity of MASLD, particularly among individuals who are overweight or obese [17,18,19]. Beyond its established role in calcium homeostasis and bone metabolism, vitamin D has been increasingly recognized for its involvement in glucose metabolism, insulin sensitivity, immune regulation, and inflammatory signaling pathways [20,21,22]. Given that many of these mechanisms are involved in the pathogenesis of MASLD, vitamin D deficiency has emerged as a potential contributor to disease progression and adverse clinical outcomes.

The recent transition from nonalcoholic fatty liver disease (NAFLD) nomenclature to metabolic dysfunction-associated steatotic liver disease reflects a conceptual shift toward recognizing the condition as a systemic metabolic disorder rather than a diagnosis defined primarily by the exclusion of alcohol use. This updated framework emphasizes the interconnected roles of metabolic dysfunction, chronic inflammation, and cardiometabolic risk factors in disease development and progression. Assessment of vitamin D status may provide additional value for risk stratification and identification of patients at increased risk for disease progression and poor clinical outcomes.

The etiology of vitamin D deficiency in CLD is likely multifactorial. Proposed mechanisms include synthetic dysfunction, decreased sunlight exposure, and inadequate dietary intake. One study reported that approximately 70% of patients with MASLD had vitamin D deficiency, compared with only 35% of healthy controls [18]. Additionally, the importance of vitamin D deficiency is underscored by its link to increased infection risk and mortality [23]. Assessing vitamin D deficiency is done by measuring 25-hydroxyvitamin D levels in the blood. Concentrations less than 20 ng/mL indicate deficiency, while levels from 20–30 ng/mL point to insufficiency [24].

Vitamin D is recognized for its immune-modulating and anti-inflammatory effects, existing mainly in two forms, D2 and D3. Vitamin D3 is produced in the skin by UV irradiation and then is metabolized in the liver and other tissues into 25-hydroxyvitamin D, the main circulating form. Vitamin D is transported by the vitamin D-binding protein (VDBP), which is synthesized by the liver. One proposed mechanism includes the dysregulation of VDBP, resulting in worsening inflammation and fibrosis [25].

Ultimately, MASLD is a prevalent, multisystem condition driven by metabolic risk factors and insulin resistance, with emerging evidence suggesting that vitamin D deficiency may exacerbate disease severity and progression.

In this study, we hypothesize that patients with MASLD who have concomitant vitamin D deficiency will have a poorer prognosis compared to those with vitamin D levels within the normal range. Consequently, this study will address the limited real-world data assessing long-term clinical outcomes in MASLD patients stratified by vitamin D status.

## 2. Materials and Methods

### 2.1. Inclusion and Exclusion Criteria

Institutional Review Board approval was obtained from the Charleston Area Medical Center. This retrospective cohort study utilized data from the TriNetX Research Network (Cambridge, MA, USA), a global federated health research platform that provides access to de-identified electronic medical record data, including demographic information, diagnoses, procedures, medications, and laboratory values. Data were extracted from the TriNetX US Collaborative Network, which comprises 171 participating healthcare organizations throughout the United States and contains longitudinal patient data spanning 2006–2025.

By utilizing this large-scale data infrastructure, we were able to capture a diverse patient population across various healthcare organizations, ensuring high external validity and the statistical power necessary to detect long-term longitudinal trends in MASLD outcomes.

**Figure 1:**
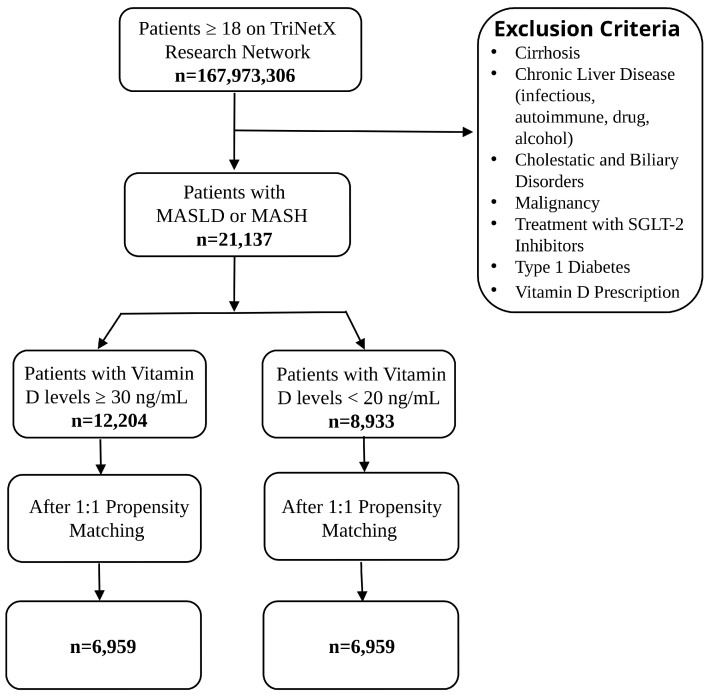
Diagram depicting inclusion and exclusion criteria for TriNetX query.

The study population included adult patients ≥ 18 years with at least one documented ICD-10-CM diagnosis code for nonalcoholic fatty liver disease (K76.0) or nonalcoholic steatohepatitis (K75.81) between 2006 and 2025. Eligible patients were required to have an available serum 25-hydroxyvitamin D laboratory measurement and at least one cardiometabolic risk factor (e.g., BMI ≥ 25 kg/m^2^, type 2 diabetes mellitus, hypertension, or hypertriglyceridemia), consistent with updated criteria for MASLD. Patients were excluded if they had cirrhosis or alternative causes of chronic liver disease, including alcohol-associated liver disease, viral hepatitis, or autoimmune hepatitis. Additional exclusion criteria included use of selected medications, such as GLP-1 receptor agonists and SGLT2 inhibitors, as well as other predefined confounders detailed in Figure 1.

Baseline demographics were extracted for each patient at the time of the index event, including age, sex, race (classified as White, Black or African American, and Other). In addition to demographic data, a comprehensive profile of pre-existing comorbidities was established using ICD-10-CM codes to capture chronic conditions that frequently co-occur with MASLD, specifically hypertension, type 2 diabetes mellitus, heart failure, coronary artery disease, chronic kidney disease (Stages 1–5) and chronic obstructive pulmonary disease.

Adult patients with MASLD or those with Metabolic-dysfunction associated steatohepatitis in the US Collaborative Network were identified. Cohorts were rigorously defined using specific clinical parameters: MASLD was identified through a combination of ICD-10-CM codes (K76.0, K75.81) and the presence of at least one metabolic risk factor, while Vitamin D status was strictly categorized based on the most recent serum 25-hydroxyvitamin D lab value prior to the index event. To ensure the integrity of the “Vitamin D Deficient” cohort, we applied a “washout” period that excluded any patients who had been prescribed Vitamin D supplements within six months prior to the index date, thereby focusing the analysis on the impact of baseline physiological deficiency. This temporal constraint was applied to minimize the confounding effects of exogenous supplementation on baseline serum levels and to isolate the impact of chronic deficiency on MASLD outcomes.

These patients were then stratified into two cohorts: (a) patients with normal vitamin D levels defined as 25-hydroxyvitamin D3 ≥ 30 ng/mL and (b) patients with vitamin D deficiency defined as 25-hydroxyvitamin D3 < 20 ng/mL (Figure 1). Patients with decompensated cirrhosis, CLDs (autoimmune, drug, alcohol, etc.), cholestatic & biliary disorders, malignancy, treatment with SGLT2, Type 1 diabetes, and vitamin D prescription are included in Figure 1. Outcomes measured included mortality rates, hospital readmissions, and ICU admissions at the 1-year and 5-year mark.

### 2.2. Statistical Analysis

To reduce the potential for selection bias and confounding inherent in retrospective electronic health record analyses, propensity score matching was performed in a 1:1 ratio using a greedy nearest-neighbor algorithm. Propensity scores were generated within the TriNetX platform using logistic regression based on baseline characteristics, including age at index event, sex, race, and relevant comorbidities such as heart failure, hypertension, chronic obstructive pulmonary disease, ischemic heart disease, and chronic kidney disease. Following matching, outcomes of interest included all-cause mortality, hospital readmissions, and ICU admissions at 1-year and 5-year follow-up intervals. Our study evaluated outcomes at a fixed follow-up time instead of examining the time until each event occurred. Therefore, Kaplan-Meier survival analysis was not included.

## 3. Results

### 3.1. Baseline Characteristics

A total of 12,204 patients with MASLD had normal serum vitamin D levels (≥30 ng/mL), while 8933 patients were classified as vitamin D deficient (<20 ng/mL). Following propensity matching, 6959 patients were included in each cohort. The mean age after propensity score matching was 46.3 ± 14.7 years in the normal vitamin D group and 46.9 ± 15.4 years in the deficient group. In both cohorts, the predominant race was White (63.5% vs. 61.6%), and hypertension was the most common comorbidity (44.9% vs. 44.0%). Additional baseline characteristics are summarized in Table 1.

### 3.2. One-Year Outcomes

**Figure 2:**
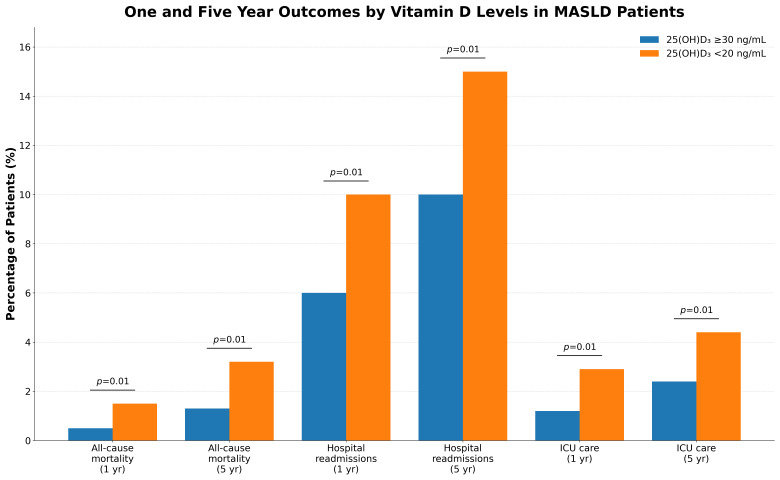
One- and five-year outcomes after propensity matching comparing patients with MASLD with vitamin D deficiency (25(OH)D_3_ < 20 ng/mL) compared to those with normal levels (25(OH)D ≥ 30 ng/mL).

Patients with MASLD and vitamin D deficiency demonstrated significantly higher rates of hospital readmissions compared to those with normal vitamin D levels (10.0% vs. 6.0%; *p* = 0.01). Similarly, ICU admission rates were greater in the vitamin D-deficient group (2.6% vs. 1.2%; *p* = 0.01). All-cause mortality was also higher among patients with vitamin D deficiency compared with those with normal levels (1.5% vs. 0.5%; *p* = 0.01). These findings are illustrated in Table 2 and Figure 2.

### 3.3. Five-Year Outcomes

At 5-year follow-up, patients with MASLD and vitamin D deficiency exhibited significantly higher rates of adverse outcomes compared to those with normal vitamin D levels. Hospital readmission rates were significantly higher among patients with vitamin D deficiency than among those with normal levels (15.0% vs. 10.0%; *p* = 0.01). ICU admission rates were also increased in the vitamin D-deficient group (4.4% vs. 2.4%; *p* = 0.01), as was all-cause mortality (3.2% vs. 1.3%; *p* = 0.01). These differences are illustrated in Table 2 and Figure 2.

## 4. Discussion

In this retrospective cohort study, patients with MASLD and vitamin D deficiency experienced significantly higher rates of hospital readmissions, ICU admissions, and all-cause mortality at both 1-year and 5-year follow-ups compared to patients who had normal vitamin D levels. These findings suggest that vitamin D deficiency may be associated with poorer prognosis in individuals with MASLD.

New guidelines by the American College of Gastroenterology recognize that although evidence of supplemental vitamin D in CLD is limited, if vitamin D level is low, supplementation may offer liver-related health benefits [26]. Though the exact benefits and mechanisms driving this remain unclear, vitamin D is well recognized for its role in calcium homeostasis and bone metabolism.

However, its relationship with inflammation remains controversial. Evidence from both in vitro and animal studies suggests that 1,25-dihydroxyvitamin D3, the most biologically active metabolite of vitamin D, exhibits strong anti-inflammatory effects by modulating the innate and adaptive immune response, thereby reducing inflammation [27,28,29]. These anti-inflammatory properties are supported by clinical observations in liver disease, where lower vitamin D levels are associated with more severe outcomes.

Emerging evidence suggests that vitamin D status may influence disease severity across a range of chronic liver diseases. In patients with chronic hepatitis B, there is an inverse correlation between vitamin D levels and severity of liver disease based on the MELD and Child-Pugh classification systems [30]. Likewise, among individuals with autoimmune hepatitis, vitamin D deficiency has been linked to an increased risk of hospitalization, hepatic decompensation, acute liver failure, and liver transplantation [31]. Collectively, these findings, together with experimental data, support a potential role for vitamin D as a modulator of hepatic inflammation, immune responses and overall disease progression, underscoring its relevance as a potential biomarker of liver disease severity.

With the prevalence of MASLD projected to approach nearly 50% of the adult population by 2050, identifying potentially modifiable risk factors is essential not only to improve clinical outcomes but also to improve the economic burden associated with the disease [7]. The estimated 10-year cost of managing MASLD and its related complications is projected to exceed $908 billion, emphasizing the urgent need for effective preventive and therapeutic strategies [32].

Interestingly, due to the observational design of this study, vitamin D may also be a surrogate marker for advanced metabolic liver disease, suggesting some temporal ambiguity. Therefore, it remains unclear whether low vitamin D levels contribute to the development of MASLD, or whether more advanced liver disease leads to lower vitamin D levels, as patients with advanced MASLD often exhibit decreased physical activity and limited sunlight exposure, alongside impaired hepatic hydroxylation of vitamin D [33].

While this observational design cannot definitively establish a causal direction of effect, the significant difference in 5-year mortality and ICU admission rates suggests that vitamin D status, regardless of whether a causal driver or a high-fidelity surrogate marker, is an essential prognostic tool for identifying MASLD patients at risk for clinical decompensation.

Despite the large sample size and statistical power provided by the TriNetX US Collaborative Network, several limitations should be considered when interpreting these findings. First, the retrospective nature of this study relies on the accuracy of ICD-10-CM coding, making it susceptible to misclassification and underreporting of MASLD and its associated metabolic comorbidities. Such coding inaccuracies may introduce information bias and affect the precision of cohort identification. Definitive diagnosis of MASLD typically requires imaging findings or histologic confirmation, which were not available in this dataset. Second, although propensity score matching was performed to balance the cohorts with respect to major demographic and clinical characteristics, residual confounding from unmeasured factors remains possible, particularly social determinants of health. Socioeconomic status, access to healthcare, food insecurity, and health literacy all may influence both vitamin D status and clinical outcomes in patients with MASLD [34].

Important factors that influence both vitamin D status and metabolic health, including dietary intake and physical activity, for example, are not comprehensively captured within the TriNetX platform and therefore could not be adjusted for in this analysis. Additionally, while patients with documented vitamin D prescriptions were excluded, over-the-counter vitamin D supplementation is common and cannot be reliably identified within the database. As a result, the true relationship between vitamin D deficiency and MASLD outcomes may be difficult to fully ascertain. Finally, although the TriNetX US Collaborative Network includes a geographically diverse patient population, the findings may not be generalizable to populations outside the United States, where genetic predisposition, environmental factors, healthcare access, and ethnic variation may influence both vitamin D status and MASLD outcomes.

## 5. Conclusions

In conclusion, vitamin D deficiency was associated with significantly worse clinical outcomes among patients with MASLD, including increased rates of hospital readmissions, ICU admissions, and all-cause mortality. These findings suggest that vitamin D status may serve as a valuable prognostic marker for risk stratification in this population. Although the observational nature of this study precluded establishing causality, our results support consideration of routine assessment of vitamin D levels in patients with MASLD to identify individuals at higher risk for adverse outcomes who may benefit from closer surveillance and targeted interventions. Further prospective studies are warranted to determine whether the correction of vitamin D deficiency can improve long-term clinical outcomes in this population.

The observed association between vitamin D deficiency and adverse outcomes is plausible given the multifaceted role of vitamin D in immune regulation, insulin sensitivity, inflammatory signaling, and hepatic fibrogenesis. Vitamin D deficiency has been linked to increased systemic inflammation, worsening metabolic dysfunction, and progression of chronic liver disease, all of which may contribute to the higher mortality observed in our cohort. As the global burden of MASLD continues to rise, identifying modifiable risk factors that can be incorporated into routine clinical practice remains an important priority. Vitamin D testing is widely available, inexpensive, and easily integrated into standard outpatient evaluation. While vitamin D supplementation alone is unlikely to address the complex pathophysiology of MASLD, recognition and treatment of deficiency may represent one aspect of a comprehensive management strategy aimed at reducing disease progression and improving patient outcomes. Future studies are needed to further clarify the mechanistic relationship between vitamin D deficiency and MASLD and to evaluate whether targeted supplementation can favorably alter the natural progression of the disease.

## Figures and Tables

**Table 1:** Baseline Characteristics of Study Cohorts Before and After Propensity Matching.

	Before Propensity Score Matching			After Propensity Score Matching		
Vitamin D Level	>30 ng/mL (*n* = 12,204)	<20 ng/mL (*n* = 8933)	Std Diff.	>30 ng/mL (*n* = 6959)	<20 ng/mL (*n* = 6959)	Std Diff.
Demographics						
Age at Index, mean years ± SD	53.5 ± 15.5	43.4 ± 16.4	0.01	46.3 ±14.7	46.9 ± 15.4	0.041
Sex						
Female	7391 (60.6)	4546 (50.9)	0.20	3773 (54.2)	3760 (54.0)	0.01
Male	4559 (37.4)	4274 (47.8)	0.21	3076 (44.2)	3092 (44.4)	0.01
Race						
White	8747 (71.7)	4888 (54.7)	0.36	4417 (63.5)	4284 (61.6)	0.04
Black or African American	706 (5.8)	1003 (11.2)	0.20	595 (8.6)	605 (8.7)	0.01
Other	1247 (10.2)	1770 (19.8)	0.27	1047 (15.0)	1125 (16.2)	0.03
Comorbidities						
Heart failure	465 (3.8)	413 (4.6)	0.04	271 (3.9)	319 (4.6)	0.03
Hypertension	6502 (53.3)	3512 (39.3)	0.28	3124 (44.9)	3065 (44.0)	0.02
Chronic kidney disease	1039 (8.5)	527 (5.9)	0.10	445 (6.4)	461 (6.6)	0.01
Chronic kidney disease—stage 5	20 (0.2)	30 (0.3)	0.03	16 (0.2)	22(0.3)	0.02
Coronary artery disease	1362 (11.2)	735(8.2)	0.10	605(8.7)	632(9.1)	0.01
Chronic obstructive pulmonary disease	538 (4.4)	334 (3.7)	0.03	269(3.9)	288 (4.1)	0.01

**Table 2:** Outcomes of patients with MASLD and normal vitamin D levels compared to patients with MASLD and vitamin D deficiency.

1-Year Outcomes	25-Hydroxy-D3 ≥ 30 ng/mL	25-Hydroxy-D3 ≤20 ng/mL	*p*	5-Year Outcomes	25-Hydroxy-D3 ≥ 30 ng/mL	25-Hydroxy-D3 ≤ 20 ng/mL	*p*
Total cases of MASLD	100% (6959)	100% (6959)	-		100% (6959)	100% (6959)	-
All-cause mortality	0.5% (38)	1.5% (102)	0.01		1.3% (87)	3.2% (221)	0.01
Hospital readmissions	6.0% (419)	10% (688)	0.01		10% (700)	15% (1013)	0.01
ICU care	1.2% (86)	2.86 (183)	0.01		2.4% (168)	4.4% (305)	0.01

## Data Availability

Data is contained within the article. The original contributions presented in this study are included in the article. Further inquiries can be directed to the corresponding author(s).

## Abbreviations

The following abbreviations are used in this manuscript:

MASLD	Metabolic dysfunction-associated steatotic liver disease
CLD	Chronic Liver Disease
VDBP	Vitamin D-binding protein
HCOs	Healthcare organizations
